# Supplementary material for: Structure–function studies of ultrahigh molecular weight isoprenes provide key insights into their biosynthesis
Source: Commun Biol. 2021 Feb 16;4:215. doi: 10.1038/s42003-021-01739-5 (PMC7887238; doi:10.1038/s42003-021-01739-5)
Supplement: Supplementary file 2 — Supplementary Information [file 42003_2021_1739_MOESM2_ESM.pdf]

Supplementary Figures and Tables

Structure-function studies of ultrahigh molecular weight isoprenes provide key insights into their biosynthesis

Hiroyuki Kajiura, Takuya Yoshizawa, Yuji Tokumoto, Nobuaki Suzuki, Shinya Takeno, Kanokwan Jumtee Takeno, Takuya Yamashita, Shun-ichi Tanaka, Yoshinobu Kaneko, Kazuhito Fujiyama, Hiroyoshi Matsumura, Yoshihisa Nakazawa

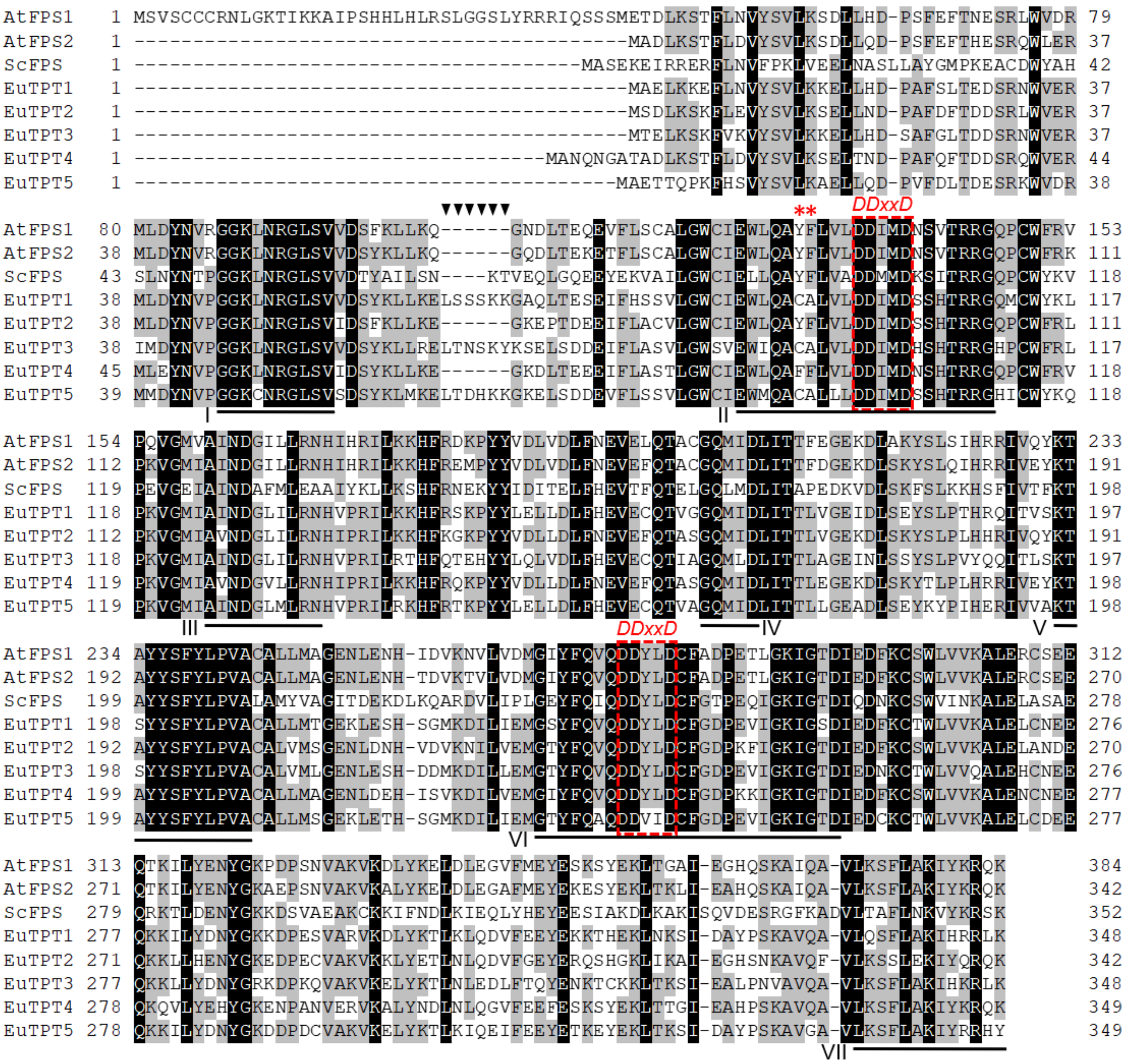

Supplementary Fig. S1 Alignment of amino acid sequences of EuTPTx, *Arabidopsis* FPS (AtFPS1 and AtFPS2), and *Saccharomyces* FPS.

The alignment was performed using CLUSTALW (http://align.genome.jp/). Identical and similar sequences are shaded in black and gray, respectively. The seven *trans*-prenyltransferase-specific motifs are underlined in black. Red dotted boxes represent DDxxD motifs. Chain length determination, upstream of the first DDxxD, are labeled with red asterisks. Triangles indicate EuTPT1, EuTPT3, and EuTPT5-specific six additional amino acids.

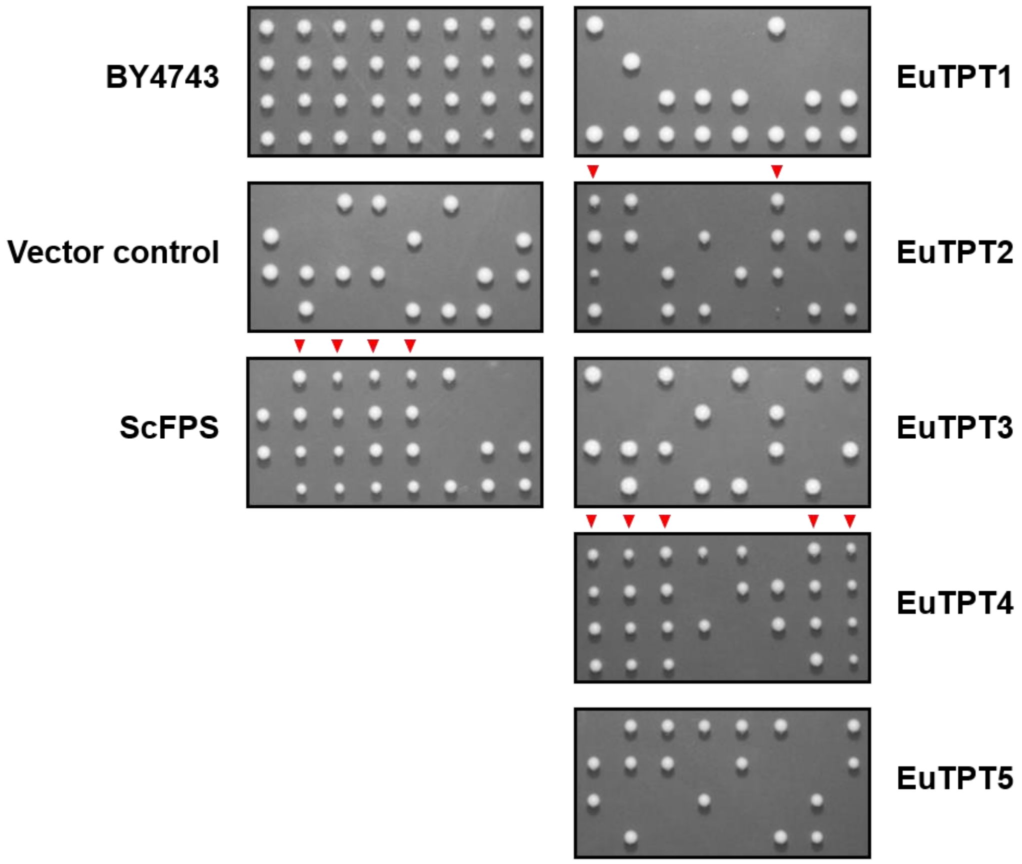

Supplementary Fig. S2 Functional characterization of EuTPTx using *S. cerevisiae Δfps* mutant. Complementation of *S. cerevisiae Δfps* was performed using yeast expression plasmids with inserting the cDNA driven under the control of *GAL1* promoter, followed by tetrad analysis. The spores were micromanipulated onto rich medium containing 2% galactose and grown for 2 days. Red triangles indicate tetrads containing two *Δfps* segregants with lethality complemented by the cDNA-expressed plasmid.

a

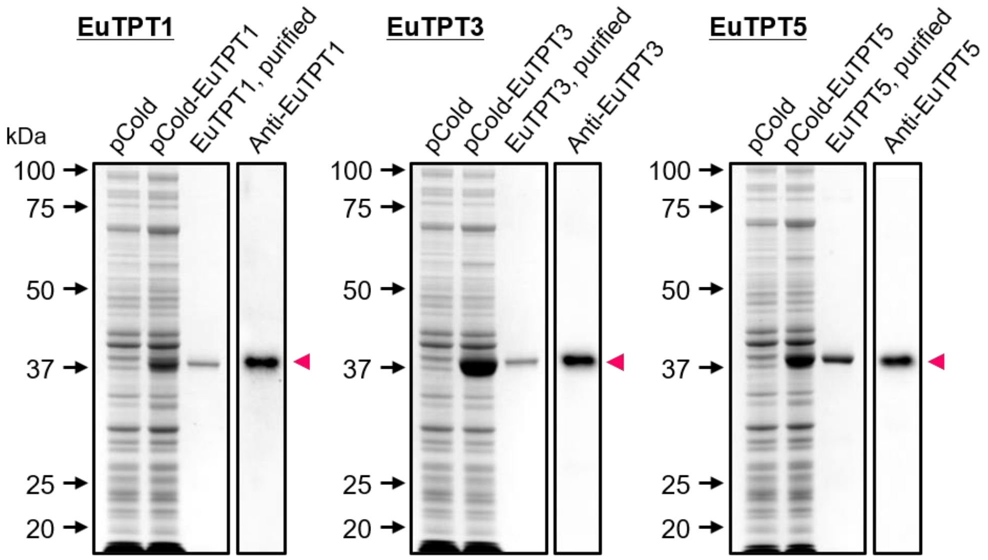

b

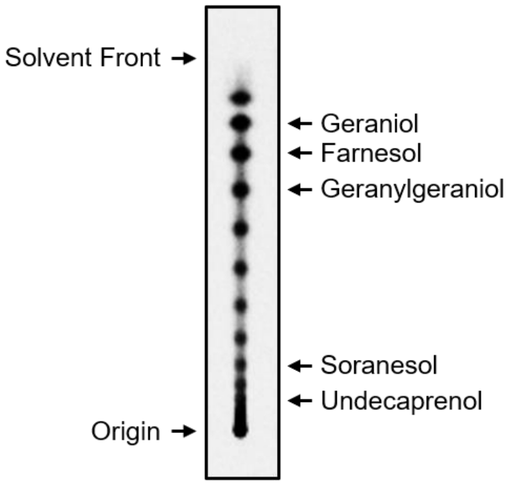

Supplementary Fig. S3 Enzymatic characterization of the recombinant EuTPTs.

(a) CBB staining and western blotting of crude and purified EuTPTs. Crude cell lysate of *E. coli* expressing EuTPTx and purified EuTPTx using Co^{2+} affinity chromatography were separated by SDS-PAGE. Triangles indicate EuTPTs.

(b) TLC analysis of the reaction product using purified EuTPT1. [1-^{14}C]IPP was incubated with EuTPT1 and FPP as allylic substrate, followed by extraction of the ^{14}C-labeled reaction products, dephosphorylation using acid phosphatase, and separation by RP-TLC. Degradation products shorter chain length of polyisoprene than primer substrate, FPP, were detected. Origin and solvent front are indicated by arrows.

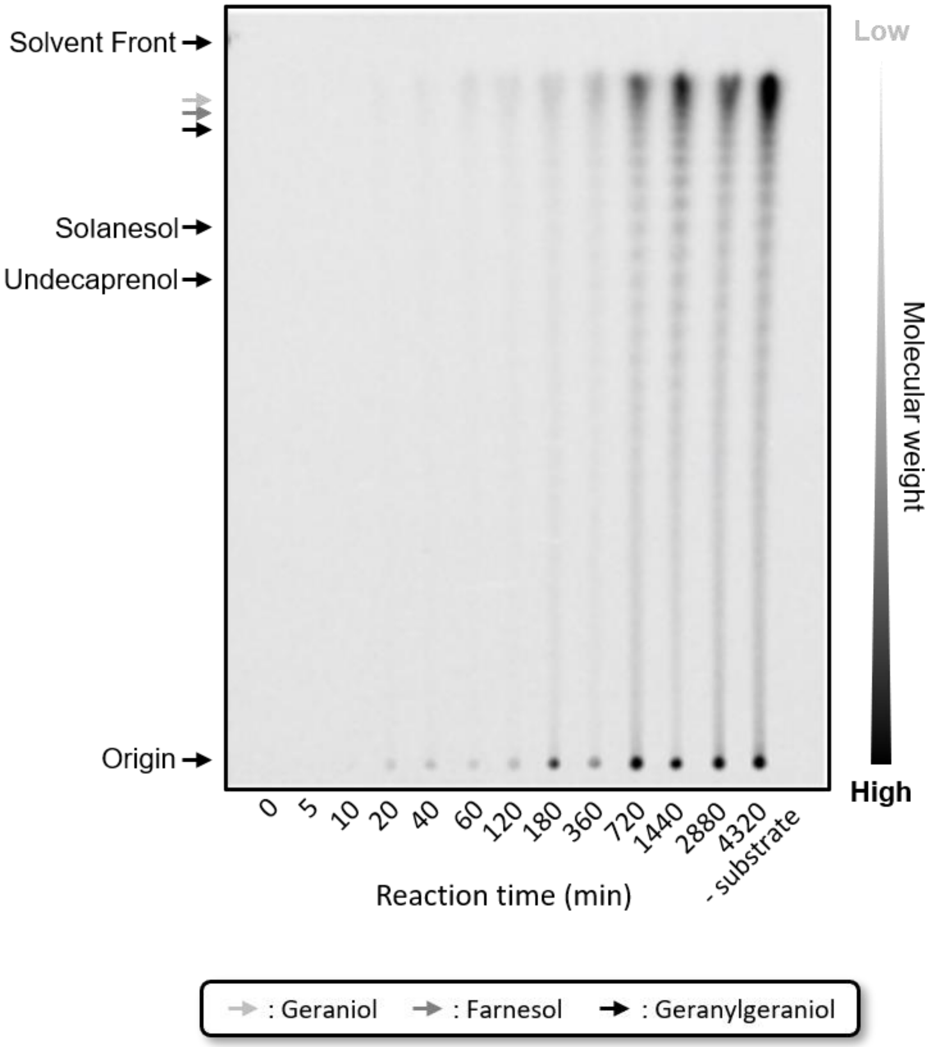

Supplementary Fig. S4 Time-dependent synthesis of ultralong-chain TPI.

The reaction products using [1-^{14}C]IPP and FPP as allylic substrate under various reaction time were separated by RP-TLC. Origin and solvent front are indicated by arrows. Geraniol, farnesol, and geranylgeraniol are indicated by light gray, gray, and black arrows, respectively.

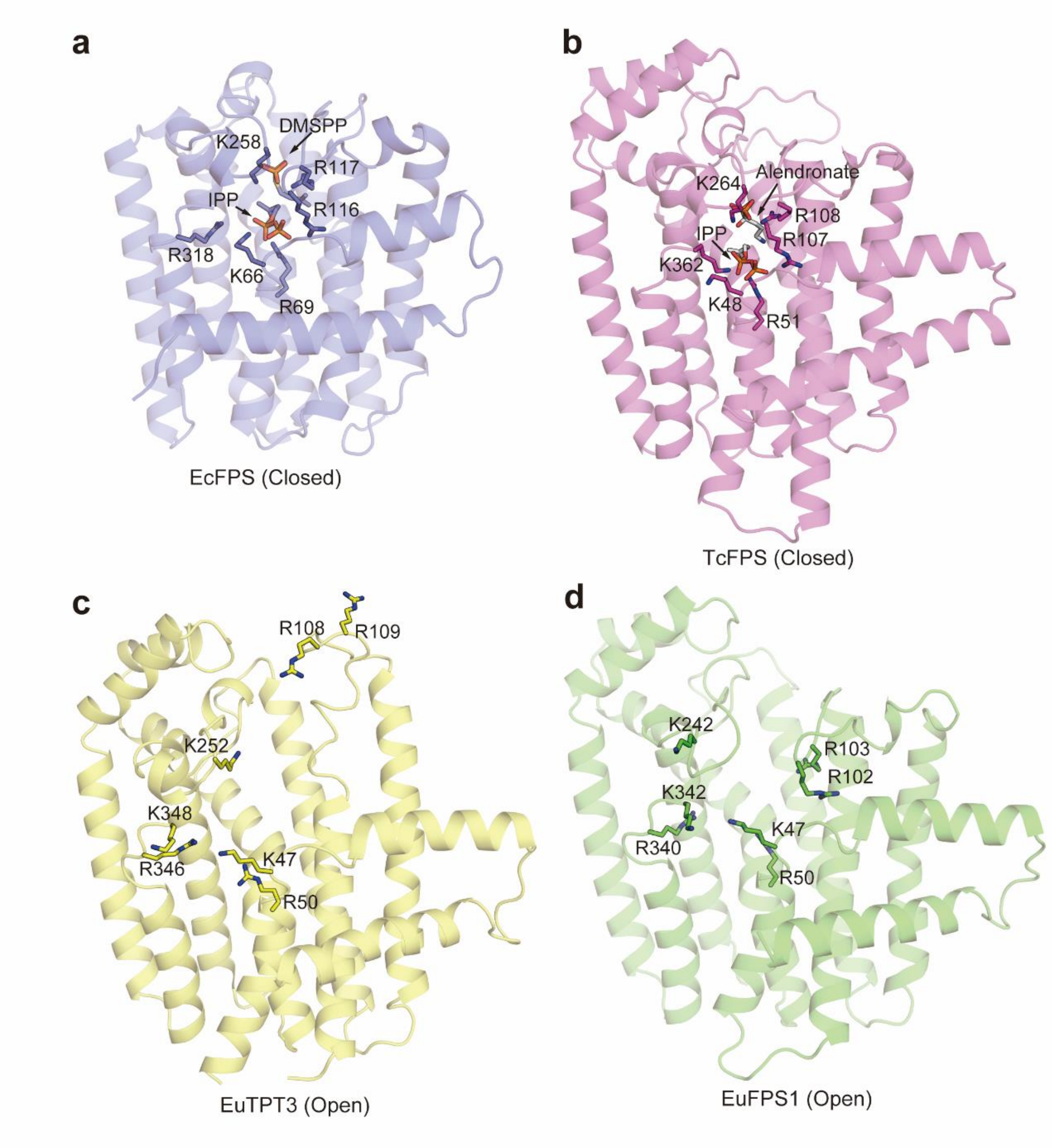

Supplementary Fig. S5 Substrate binding of FPSs.

(a) FPS from *Escherichia coli* in complex with IPP and DMSPP (PDB code 1RQI).

(b) FPS from *Trypanosoma cruzi*[1] in complex with IPP and Alendronate (PDB code 1YHM). Substrates and basic residues contributing substrate binding are shown by stick model.

(c) Basic residues of EuTPT3 presumably involved in substrate binding are shown by stick model.

(d) Basic residues of EuFPS1 presumably involved in substrate binding are shown by stick model.

1. Gabelli, S.B., McLellan, J.S., Montalvetti, A., Oldfield, E., Docampo, R. & Amzel, L.M. Structure and mechanism of the farnesyl diphosphate synthase from *Trypanosoma cruzi*: implications for drug design. *Proteins*, **62**, 80, (2006).

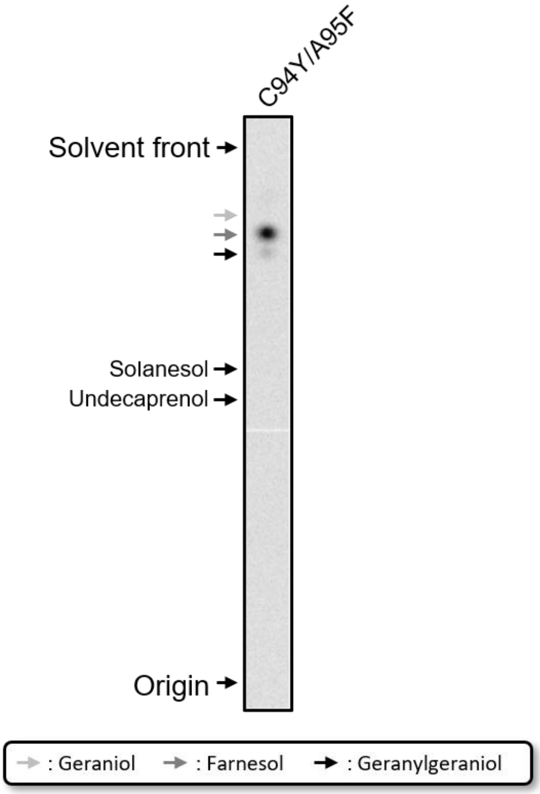

Supplementary Fig. S6 Analysis of the reaction product of EuTPT3(C94Y/A95F) mutant

The reaction products of EuTPT3(C94Y/A95F) mutant using [1-^{14}C]IPP and GPP as allylic substrate were separated by RP-TLC. Origin and solvent front are indicated by arrows. Geraniol, farnesol, and geranylgeraniol are indicated by light gray, gray, and black arrows, respectively.

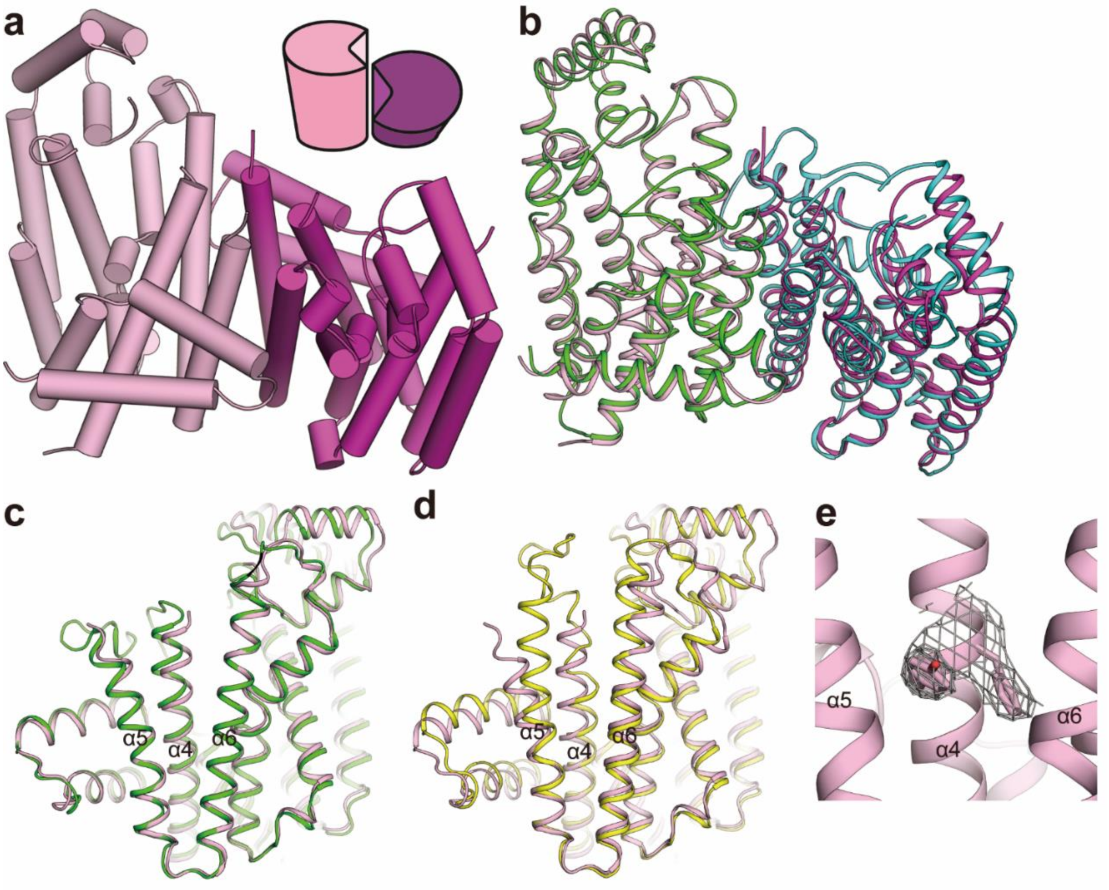

Supplementary Fig. S7 Structure of EuTPT3(C94Y/A95F) mutant.

(a) Overall structures of EuTPT3(C94Y/A95F) mutant shown by cartoon cylinder model. Chain A and B are colored in light pink and purple, respectively.

(b) Super imposed dimeric structures of EuFPS1(C94Y/A95F) mutant (light pink and purple) and EuFPS1 (green and cyan). Structures are shown by cartoon ribbon models. (c) Super imposed subunit structures of EuTPT3(C94Y/A95F) (pink) and EuFPS1 (green).

(d) Super imposed subunit structures of EuTPT3(C94Y/A95F) mutant (pink) and EuTPT3 wild-type (yellow). E) Close-up view of mutated residues of EuTPT3. Mutated residues are shown by stick model. 2Fo - Fc map (1.0 σ) around the residues is overlaid the stick model.

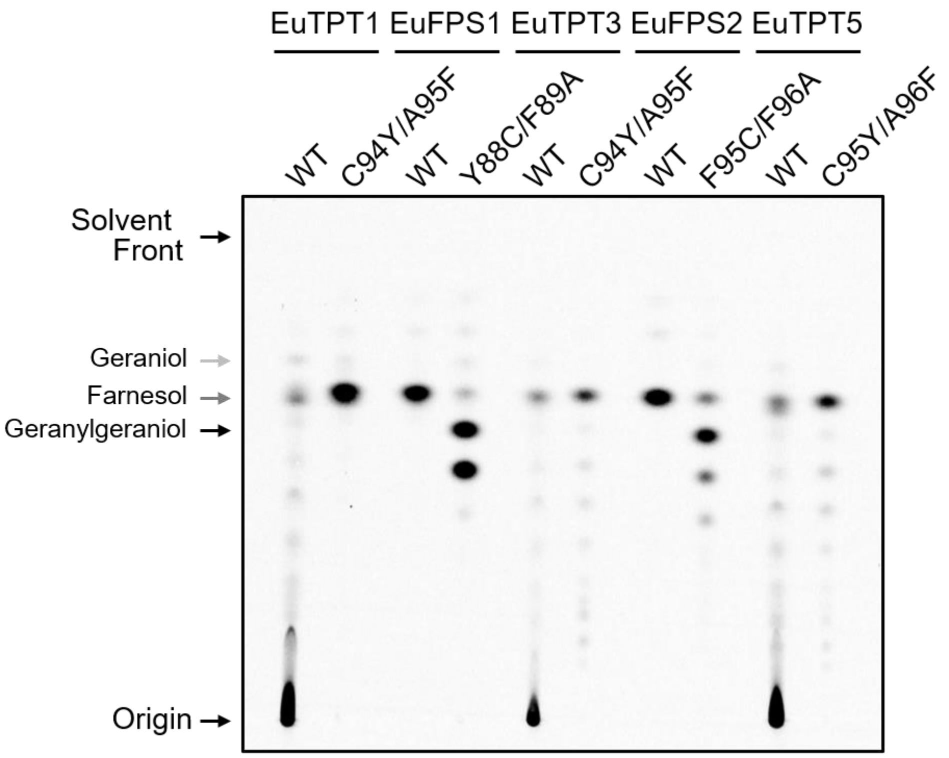

Supplementary Fig. S8 Analysis of the reaction products of EuTPTs mutant.

The reaction products of WT and amino acid-replaced EuTPT1, EuTPT2 (EuFPS1), EuTPT3, EuTPT4 (EuFPS2), and EuTPT5 using [1-^{14}C]IPP and GPP as allylic substrate were separated by RP-TLC. Origin and solvent front are indicated by arrows. Geraniol, farnesol, and geranylgeraniol are indicated by light gray, gray, and black arrows, respectively.

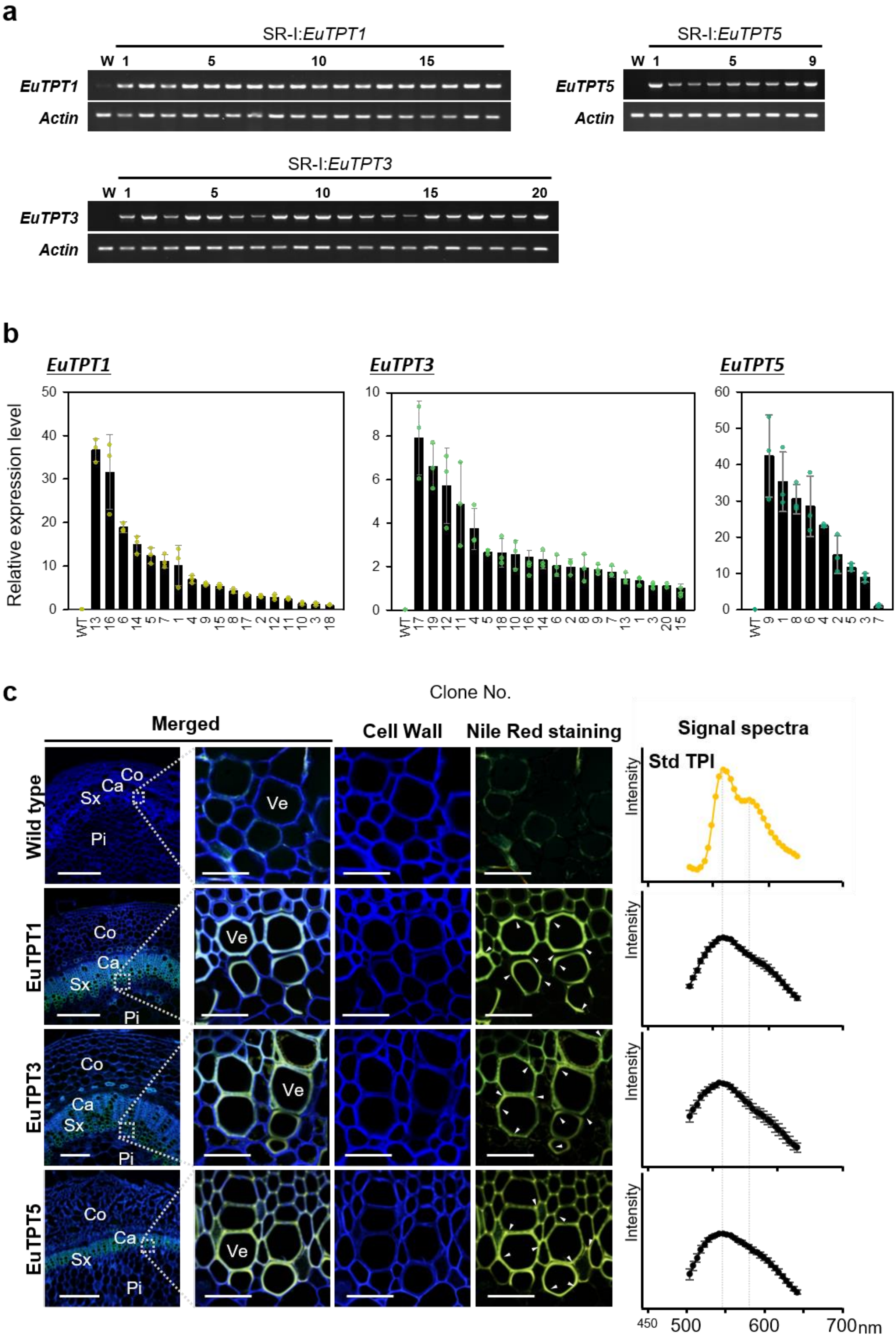

a
SR-I:EuTPT1
W 1 5 10 15
EuTPT1
Actin
SR-I:EuTPT5
W 1 5 9
EuTPT5
Actin
SR-I:EuTPT3
W 1 5 10 15 20
EuTPT3
Actin
b
EuTPT1
EuTPT3
EuTPT5
Relative expression level
WT 13 16 6 14 5 7 1 4 9 15 8 17 2 12 11 10 3 18
WT 17 19 12 11 4 5 18 10 16 14 6 2 8 9 7 13 1 3 20 15
WT 9 1 8 6 4 2 5 3 7
Clone No.
c
Merged
Cell Wall
Nile Red staining
Signal spectra
Wild type
EuTPT1
EuTPT3
EuTPT5
Co
Ca
Sx
Pi
Ve
Std TPI
Intensity
450 500 600 700nm

Supplementary Fig. S9 Selection of the transformant expressing the highest *EuTPTx* and their phenotype.

(a) RT-PCR analysis of *EuTPTx* expressed in tobacco transformants. The expression was confirmed using cDNA as a template. *Actin* was used as a control. W: wild type; SR-I:*EuTPTx*: tobacco SR-I plant expressing *EuTPTx*.

(b) qRT-PCR analysis of transcript levels of *EuTPTx* expressed in tobacco transformants. The transcript levels were normalized against tobacco *glyceraldehyde-3-phosphate dehydrogenase* gene and calculated in comparison with that of the transformant showing lowest expression level. The templates were prepared from biologically independent three plants of the same clone. Standard deviation was used to calculate error bars.

(c) Histochemical staining of transgenic tobacco plant expressing EuTPTx. (Left) The section prepared from 4-week-old plant stem was stained with Nile red and calcofluor to visualize polyisoprene and cell wall, respectively. (Right) The fluorescence spectra of the stained region of interest were measured using spectral confocal laser scanning microscopy and averaged. White triangles indicate target region for the acquisition of the fluorescence spectra. Bars in the overview of the stem and in the enlarged view are 500 μm and 50 μm, respectively. Ca, cambium; Co, cortex; Pi, pith; Sx, secondary xylem; Ve, vessel.

a

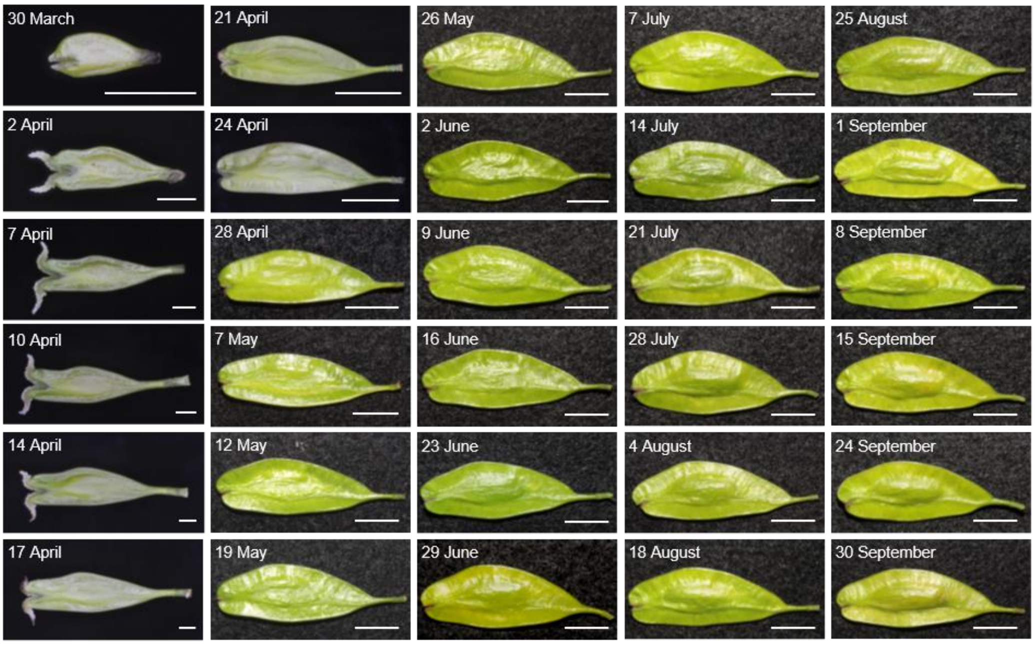

b

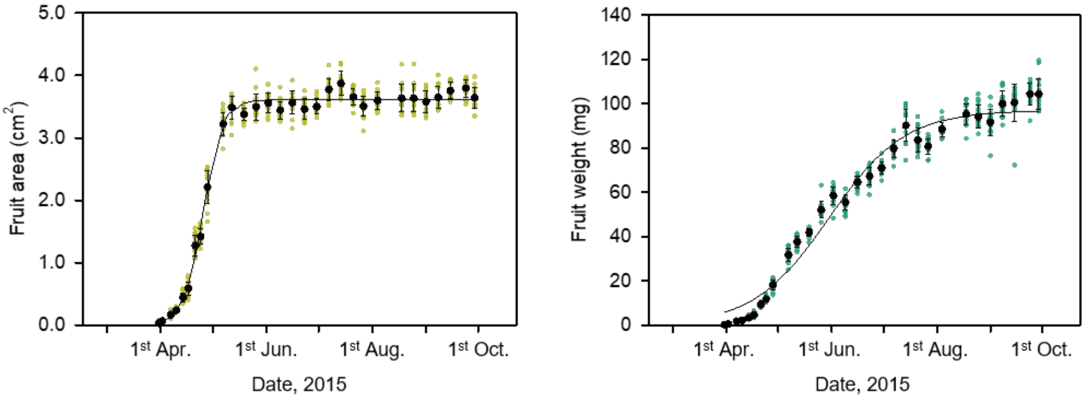

Supplementary Fig. S10 Seasonal changes of *Euccomia* fruit.

(a) *Euccomia* fruit development in 2015. Bars are 2 mm from March 30 to April 17; 10 mm from April 21.

(b) *Eucommia* fruit area and weight in 2015. Logarithmic growth curves for fruit area and weight are shown. Regression equations are as followes: Fruit area (cm^2), $Y = 3.61/[1 + 100.93*exp^{-0.17*(\text{Date from 31 March})}]$, $r^2 = 0.9825$; Fruit weight (mg), $Y = 96.92/[1 + 16.43*exp^{-0.05*(\text{Date from 31 March})}]$, $r^2 = 0.9657$. All r^2 values of fruit area and fruit weight were over 0.9. All the values were analyzed in biologically independent samples (*n* = 13-15) and the averages are shown as black symbols.

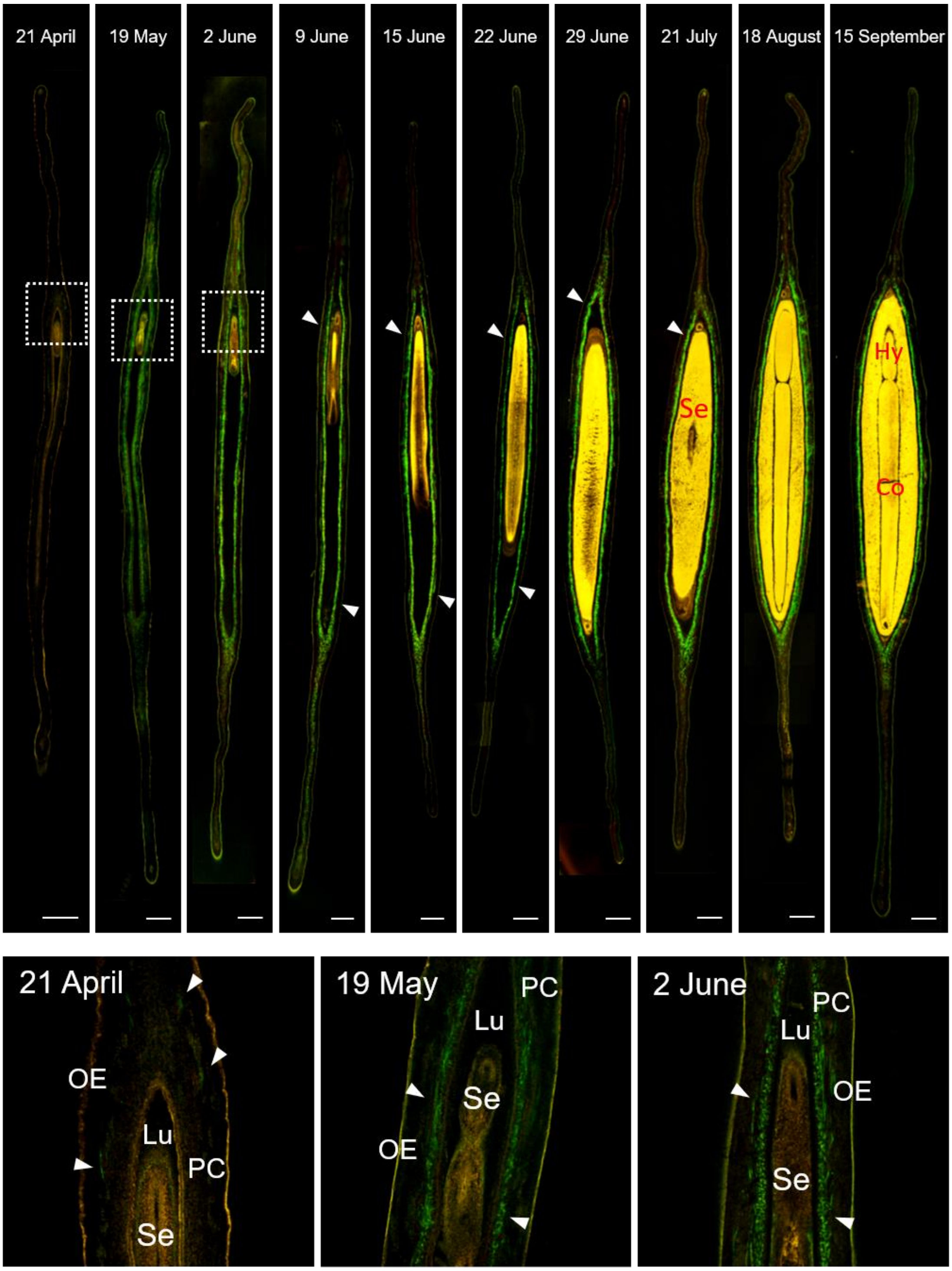

Supplementary Fig. S11 Histochemical staining of *Eucommia* fruit.

(Top) Sections of *Eucommia* fruit (XY direction, shown in Fig. 6b) were stained with Nile red. Unmixed images of TPI (green), seed (yellow, orange), and lipid (red) were photomerged. (Bottom) close-up views of the upper images shown in dotted white boxed areas. White triangles indicate TPI. OE, outer epidermis; Lu, lumen; PC, parenchyma cells; Se, seed; Hy, hypocotyl; Co, cotyledon. Bars: 1 mm.

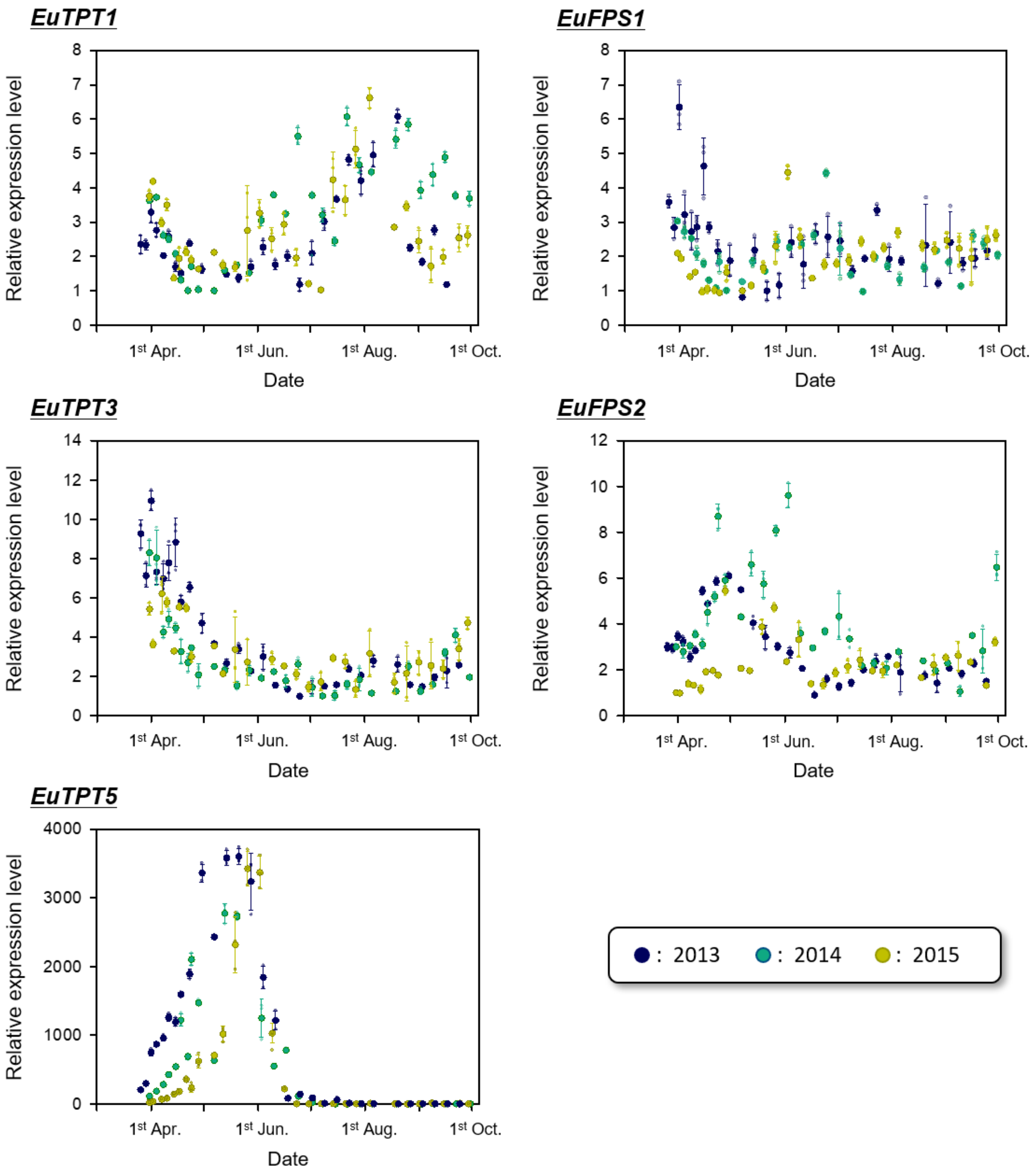

Supplementary Fig. S12 Seasonal expression profile of *EuTPTs*.

qRT-PCR analysis of transcript levels of *EuTPTs* were performed using cDNA prepared from *Eucommia* fruits. Transcript levels were normalized using *ACTIN* and calculated in comparison with that of the sample of the lowest expression level. The templates were prepared from independent triplicates. Dark blue, green, and yellow circles indicate the transcript levels of *EuTPTs* in 2013, 2014, and 2015, respectively. All expression levels were analyzed in independent samples (n = 3).

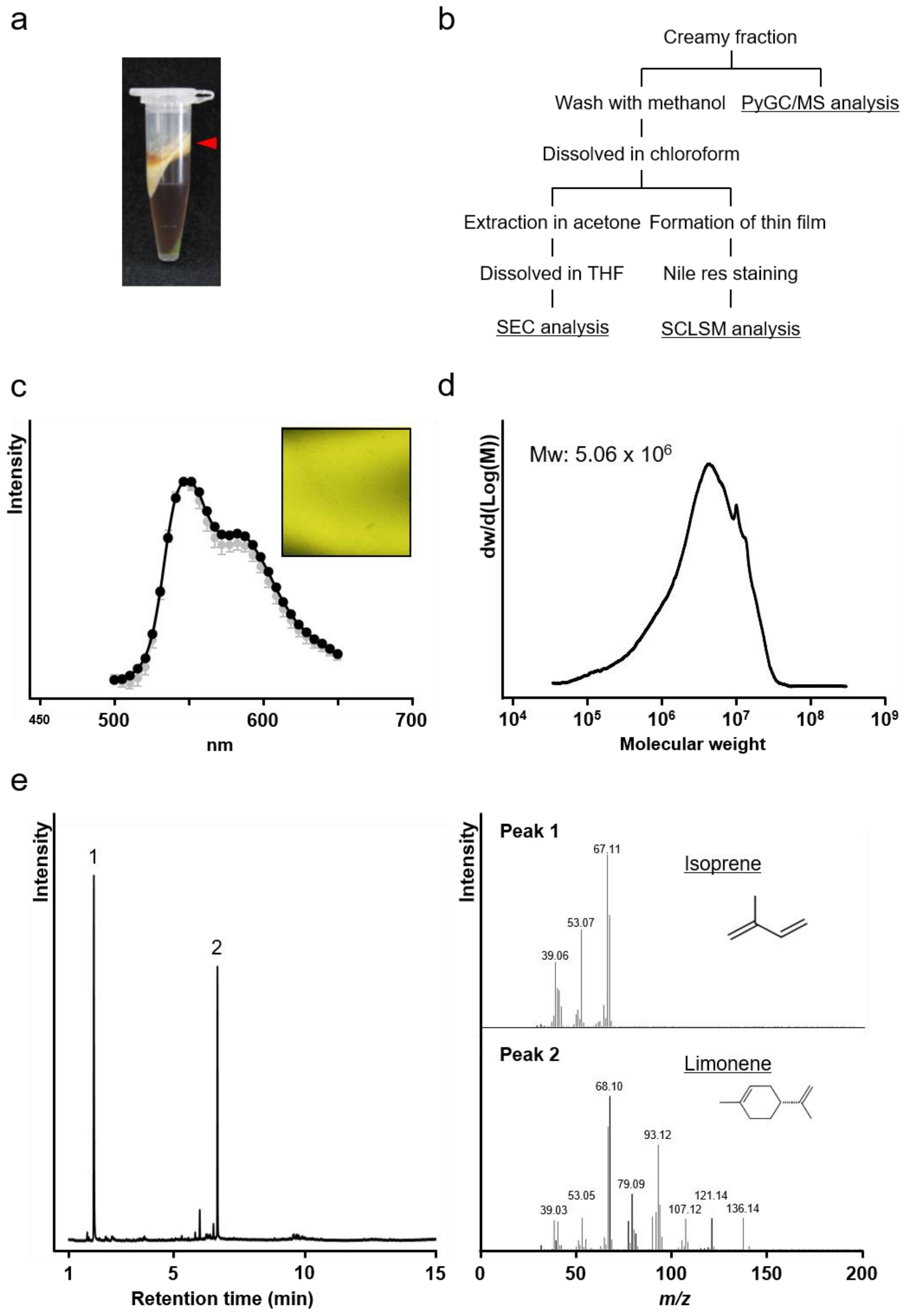

a
b
Creamy fraction
Wash with methanol
PyGC/MS analysis
Dissolved in chloroform
Extraction in acetone
Formation of thin film
Dissolved in THF
Nile res staining
SEC analysis
SCLSM analysis
c
Intensity
450
500
600
700
nm
d
dw/d(Log(M))
Mw: 5.06 x 10^6
10^4
10^5
10^6
10^7
10^8
10^9
Molecular weight
e
Intensity
1
2
1
5
10
15
Retention time (min)
Intensity
Peak 1
Isoprene
67.11
53.07
39.06
Peak 2
Limonene
68.10
93.12
79.09
53.05
121.14
39.03
107.12
136.14
0
50
100
150
200
m/z

Supplementary Fig. S13 Physicochemical analysis of washed polyisoprene.

(a) TPI layer after centrifugation. Red triangle indicates TPI prepared from *Eucommia* pericarp collected on 26 May, 2015.

(b) Schematic representation of the preparation of TPI for each analysis.

(c) Fluorescence spectra of TPI and commercially available *trans*-polyisoprene stained with Nile red. Small insets show the rubber film stained with Nile red and used to acquire the fluorescence spectra. Black: TPI; gray: commercial *trans*-polyisoprene.

(d) Molecular weight distribution of washed TPI.

(e) Pyrogram of washed TPI and molecular mass of two predominant signals in PyGC analysis. The pyrolysates from washed TPI, isoprene and limonene, were identified using NIST MS search 2.2.

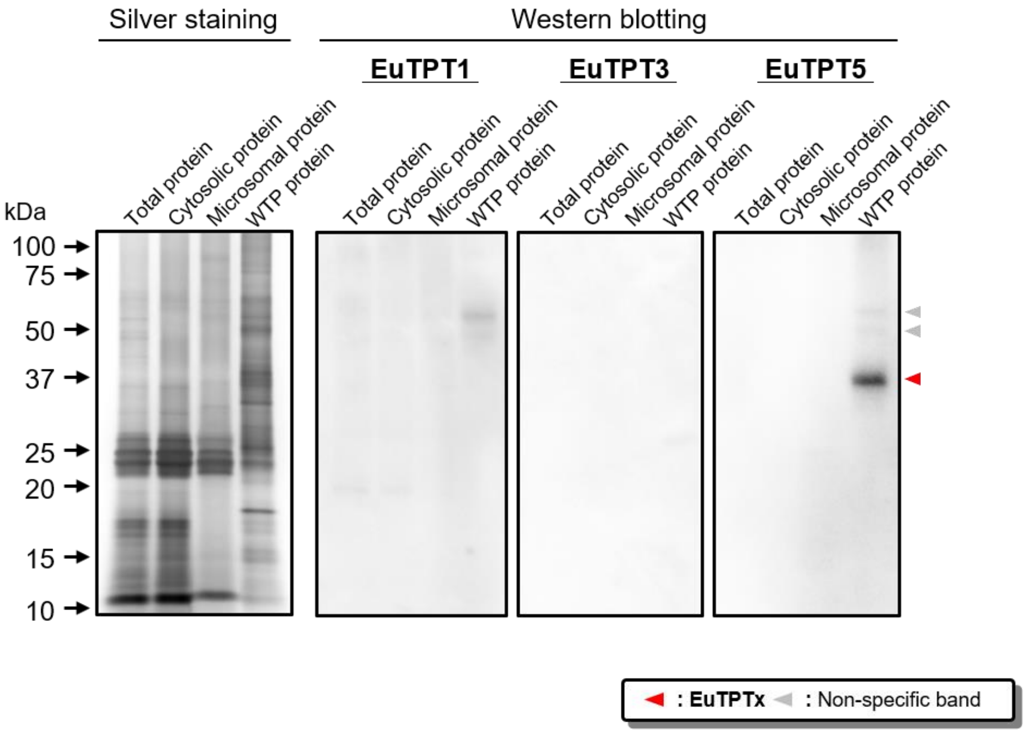

Supplementary Fig. S14 Localization analysis of EuTPTx.

Western blotting analysis of EuTPTs using total protein, cytosolic protein, microsomal protein, and washed TPI protein (WTP). Red and gray triangles indicate immuno-active and non-specific bands, respectively.

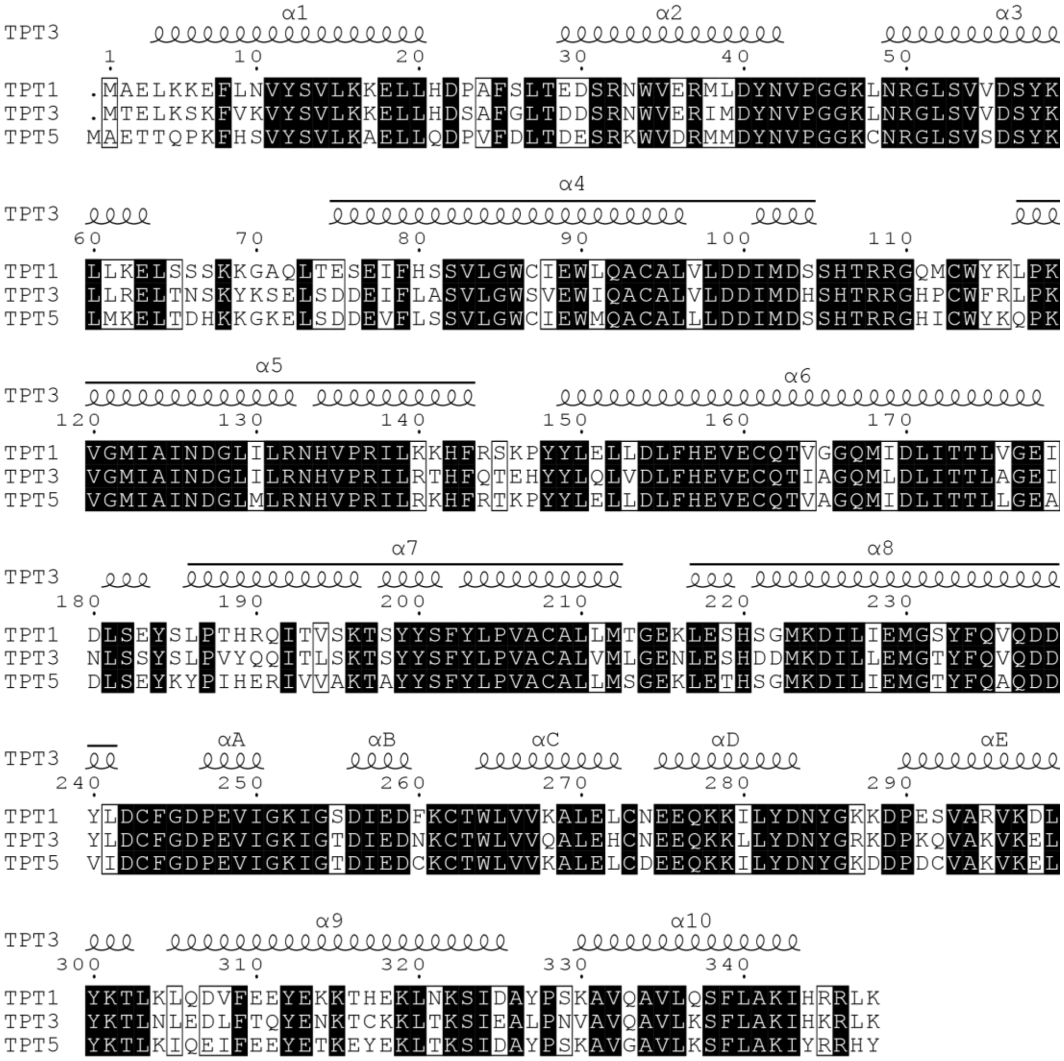

Supplementary Fig. S15 Multiple sequence alignment of EuTPT1, EuTPT3 and EuTPT5.

The alignment was performed using CLUSTALW (http://align.genome.jp/). Identical and similar sequences are shaded in black and boxed, respectively. The secondary structure of EuTPT3 is shown above the sequences.

a

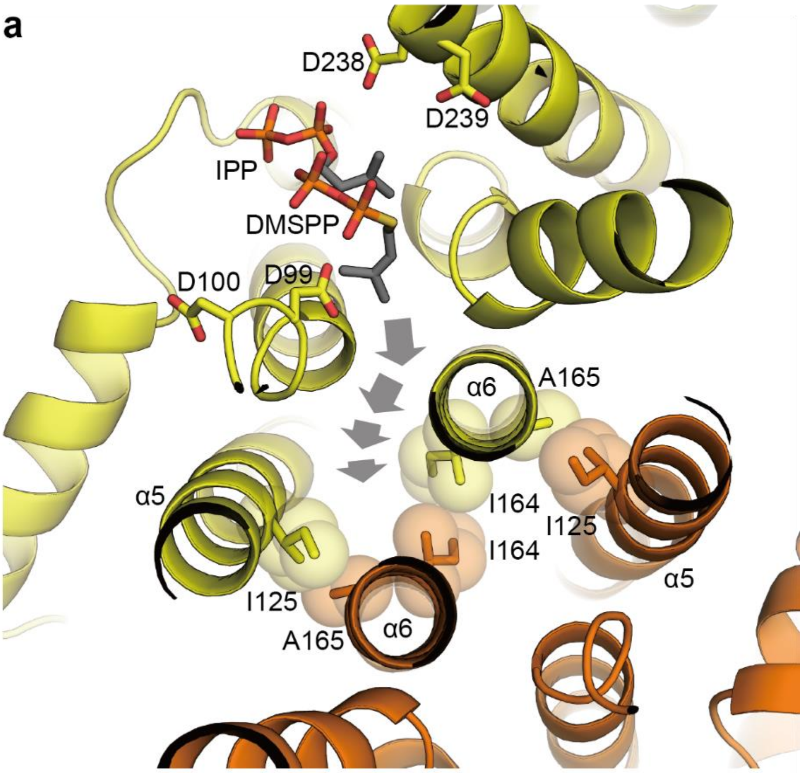

b

```
TPT1 160 ECQTVGGQMID 170
TPT3 160 ECQTIAGQMLD 170
TPT5 161 ECQTVAGQMID 171
```

Supplementary Fig. S16 Interactions of hydrophobic residues in the tunnel of EuTPT3.

(a) Top view of the substrate binding pocket and the tunnel of EuTPT3. DMSPP and IPP are placed by superimposition of the EcFPS-IPP complex (PDB code 1RQI). Putative path for the product is indicated by gray arrows. Hydrophobic residues forming contacts in the tunnel are shown as spheres.

(b) Amino acid sequence alignment of Ile164-Ala165 in EuTPTP3 with EuTPT1 and EuTPT5.

Supplementary Table S1 Enzyme properties of EuTPTs

	EuTPT1	EuTPT3	EuTPT5
**Optimum pH**	7.0-8.0 (Highest activity: pH 7.5)	7.0-8.5 (Highest activity: pH 7.5)	6.0-8.0 (Highest activity: pH 7.5)
**Optimum temperature**	20-35˚C (Highest activity: 35˚C)	20-40˚C (Highest activity: 35˚C)	20-40˚C (Highest activity: 30˚C)
**Metal ion dependency**			
Ca^{2+}	23.4±1.2%	12.9±2.4%	15.0±0.4%
Co^{2+}	100 ±11.7%	100 ±3.6%	100 ±0.5%
Mg^{2+}	72.6±1.0%	72.8±0.9%	99.4±3.0%
Mn^{2+}	95.2±1.6%	90.0±4.9%	95.1±2.4%
Ni^{2+}	25.5±0.7%	20.3±0.4%	32.1±1.6%
Zn^{2+}	0.3±0.1%	1.6±0.3%	0.8±0.2%
-	19.5±1.0%	11.6±0.7%	24.6±0.7%

The relative activities in metal-ion dependency were calculated on the basis of the addition of metal ion of Co^{2+} (100%).
