# Supplementary material for: Structure–function studies of ultrahigh molecular weight isoprenes provide key insights into their biosynthesis
Source: Commun Biol. 2021 Feb 16;4:215. doi: 10.1038/s42003-021-01739-5 (PMC7887238; doi:10.1038/s42003-021-01739-5)
Supplement: Supplementary file 3 — Description of Additional Supplementary Files [file 42003_2021_1739_MOESM3_ESM.pdf]

Description of Additional Supplementary Files

File Name: Supplementary Data 1

Description: Numerical data used to build the plots in figures 1c and 6a.

File Name: Supplementary Data 2

Description: Oligonucleotides for PCR and qRT-PCR used in this study.
