# Supplementary material for: Structure–function studies of ultrahigh molecular weight isoprenes provide key insights into their biosynthesis
Source: Commun Biol. 2021 Feb 16;4:215. doi: 10.1038/s42003-021-01739-5 (PMC7887238; doi:10.1038/s42003-021-01739-5)
Supplement: Supplementary file 6 — Reporting Summary [file 42003_2021_1739_MOESM6_ESM.pdf]

nature research

Corresponding author(s): Hiroyoshi MATSUMURA

Last updated by author(s): Dec 9, 2020

Reporting Summary

Nature Research wishes to improve the reproducibility of the work that we publish. This form provides structure for consistency and transparency in reporting. For further information on Nature Research policies, see our Editorial Policies and the Editorial Policy Checklist.

Statistics

For all statistical analyses, confirm that the following items are present in the figure legend, table legend, main text, or Methods section.

n/a	Confirmed	
☐	☒	The exact sample size (*n*) for each experimental group/condition, given as a discrete number and unit of measurement
☐	☒	A statement on whether measurements were taken from distinct samples or whether the same sample was measured repeatedly
☒	☐	The statistical test(s) used AND whether they are one- or two-sided<br>*Only common tests should be described solely by name; describe more complex techniques in the Methods section.*
☒	☐	A description of all covariates tested
☒	☐	A description of any assumptions or corrections, such as tests of normality and adjustment for multiple comparisons
☐	☒	A full description of the statistical parameters including central tendency (e.g. means) or other basic estimates (e.g. regression coefficient) AND variation (e.g. standard deviation) or associated estimates of uncertainty (e.g. confidence intervals)
☒	☐	For null hypothesis testing, the test statistic (e.g. *F*, *t*, *r*) with confidence intervals, effect sizes, degrees of freedom and *P* value noted<br>*Give P values as exact values whenever suitable.*
☒	☐	For Bayesian analysis, information on the choice of priors and Markov chain Monte Carlo settings
☒	☐	For hierarchical and complex designs, identification of the appropriate level for tests and full reporting of outcomes
☐	☒	Estimates of effect sizes (e.g. Cohen's *d*, Pearson's *r*), indicating how they were calculated

Our web collection on statistics for biologists contains articles on many of the points above.

Software and code

Policy information about availability of computer code

Data collection	BSS (Crystallographic data collection at SPring-8)
Data analysis	EZ-C1 3.40 software (Nikon), Adobe Photoshop CS4, R ver. 3.2.3., StepOne Software ver.2.1, XDS, Phenix , Coot and PyMol were used for data analysis.

For manuscripts utilizing custom algorithms or software that are central to the research but not yet described in published literature, software must be made available to editors and reviewers. We strongly encourage code deposition in a community repository (e.g. GitHub). See the Nature Research guidelines for submitting code & software for further information.

Data

Policy information about availability of data

All manuscripts must include a data availability statement. This statement should provide the following information, where applicable:

- Accession codes, unique identifiers, or web links for publicly available datasets
- A list of figures that have associated raw data
- A description of any restrictions on data availability

The dataset underlying the findings are available from the corresponding author on reasonable request. Coordinates and structure factors of EuTPT3 WT form 1, EuTPT3 WT form 2, EuTPT3 (C94Y/A95F) and EuFPS1 have been deposited in the Protein data bank (PDB codes: 7BUU, 7BUV, 7BUW and 7BUX).

Field-specific reporting

Please select the one below that is the best fit for your research. If you are not sure, read the appropriate sections before making your selection.

[x] Life sciences   [ ] Behavioural & social sciences   [ ] Ecological, evolutionary & environmental sciences

For a reference copy of the document with all sections, see nature.com/documents/nr-reporting-summary-flat.pdf

Life sciences study design

All studies must disclose on these points even when the disclosure is negative.

Sample size	No statistical method was used to predetermine sample size, but widely used sample sizes of 3 or more were used for morphological analysis of Eucommia ulmoides pericarp samples. All enzymatic activities of recombinant proteins were also repeated at least 3 times to obtain mean +/- STDEV values. Relative gene expression levels in Eucommia ulmoides pericarp and transgenic plants were analyzed using three independent samples in the same clone to obtain mean +/- STDEV values.
Data exclusions	No data were excluded from the analyses.
Replication	Analysis of the recombinant enzymatic analysis (Fig. 1, Supplementary Table S1), localization analysis (Fig. 2), expression analysis (Supplementary Fig. S12), histochemical analysis (Fig. 6, Supplementary Fig. S9, Supplementary Fig. S11), morphological analysis (Fig. 6, Supplementary Fig. S10) and tetrad analysis (Supplementary Fig. S2) were replicated.
Randomization	All analyses using Eucommia ulmoides pericarp, pericarps were randomly selected in the temporal collection of Eucommia ulmoides pericarps.
Blinding	Blinding experiments were not necessary as experimenters operated according to standard methods, analyses of all data, and obtained scientific conclusions. There was no personal preference for the experimental subjects and results.

Reporting for specific materials, systems and methods

We require information from authors about some types of materials, experimental systems and methods used in many studies. Here, indicate whether each material, system or method listed is relevant to your study. If you are not sure if a list item applies to your research, read the appropriate section before selecting a response.

Materials & experimental systems

n/a	Involved in the study	
[ ]	[x]	Antibodies
[x]	[ ]	Eukaryotic cell lines
[x]	[ ]	Palaeontology and archaeology
[x]	[ ]	Animals and other organisms
[x]	[ ]	Human research participants
[x]	[ ]	Clinical data
[x]	[ ]	Dual use research of concern

Methods

n/a	Involved in the study	
[x]	[ ]	ChIP-seq
[x]	[ ]	Flow cytometry
[x]	[ ]	MRI-based neuroimaging

Antibodies

Antibodies used	All anti-EuTPTs antibodies were obtained from Sigma or GeneDesign (Osaka, Japan) using polyclonal antibody production services (1/2000 dilution in recombinant protein detection and 1/100 dilution in immunostaining).
Validation	The antibodies were produced using the peptide at Lys62-Phe79 in EuTPT1, Leu61-Asp75 in EuTPT3, and Lys60-Ser75 in EuTPT5.
